# SIRT-ain relief from age-inducing stress

**DOI:** 10.18632/aging.100283

**Published:** 2011-02-08

**Authors:** Dan Zhang, Yufei Liu, Danica Chen

**Affiliations:** ^1^ Department of Nutritional Science & Toxicology, University of California, Berkeley, CA 94720, USA; ^2^ Department of Molecular & Cell Biology, University of California, Berkeley, CA 94720, USA

**Keywords:** Aging, cancer, sirtuins, Sirt3, p53, ROS

## Abstract

Aging is one of the most fundamental biological processes. It results in a decline in physiological function and an increased risk for pernicious diseases such as cancer. Oxidative stress has been proposed as a major cause of aging, but experimental tests of this hypothesis have been discouraging. Calorie restriction (CR) prevents age-related decline, but there are still gaps in our knowledge of the exact mechanisms underlying this feat. Finally, a tenuous balance exists between aging and cancer, calling for a search for interventions that prevent both aging and cancer. Recent work on the mammalian sirtuin SIRT3 has shed light on these long-standing issues and suggested new approaches to ameliorate the ravages of aging.

## Oxidative Stress and Aging

How does aging occur? Can we delay the aging process? These are questions that have been asked for hundreds if not thousands of years. Many hypotheses have been proposed, but the majority of them have failed to stand up to further scrutiny. In 1956, Harman proposed the oxidative stress theory of aging, which has gained great popularity among aging researchers [1]. It postulates that the production of reactive oxygen species (ROS) is the major determinant of lifespan. ROS can be produced in multiple compartments within cells, but mitochondria account for over 90% of ROS generation [2]. Counteracting cellular ROS are antioxidant enzymes such as superoxide dismutases (SODs). The level of cellular ROS is determined by the balance between the generation of ROS and its detoxification.

The oxidative stress theory is supported by the observation that the levels of oxidative stress and damage increase with age in a wide variety of animal models [3]. Also, longer-lived animals tend to have reduced oxidative damage and/or increased resistance to oxidative stress. However, under experimental settings, the theory has met with some difficulties. Studies in mice where the expressions of antioxidant enzymes are genetically modified call into serious question the oxidative stress theory of aging [4]. Although deleting the antioxidant genes has deleterious effects, surprisingly, under or overexpressing a number of genes encoding antioxidants mostly has no effect on lifespan. Even mice that are transgenic for SOD2, a crucial mitochondrial antioxidant, do not show an increase in lifespan. Is the connection between oxidative stress and lifespan simply a coincidence? Is it time to throw away the oxidative stress theory of aging?

A potential explanation for this controversy has emerged from recent studies on the mammalian mitochondrial deacetylase SIRT3. Endogenous SOD2 is modified by acetylation. By deacetylating specific lysine residues on SOD2, SIRT3 improves the ability of SOD2 to scavenge for ROS [5,6]. Interestingly, increasing SOD2 expression alone only modestly reduces cellular ROS. However, when SOD2 overexpression is combined with SIRT3 overexpression, the reduction in cellular ROS is much greater. These observations suggest that only altering SOD2 expression is insufficient to significantly reduce cellular oxidative stress, and that it needs to be modified by deacetylation to operate at peak activity. In addition to SOD2, a number of antioxidants such as SOD1 and glutathione peroxidase have been shown to be acetylated [7]. It is possible that these antioxidants are also regulated via deacetylation.

Perhaps the oxidative stress theory of aging has not been given a fair trial. These latest findings suggest that future tests of the theory need to take into account not only the expression level of antioxidants, but their activity level as well. In the case of SOD2, generating SOD2 transgenic mice is not enough, as SOD2 requires SIRT3 to become fully active. Thus, an interesting and pivotal test of the oxidative stress theory of aging will be whether mice that are transgenic for both SOD2 and SIRT3 have extended lifespans. In addition to having major implications for the oxidative stress theory of aging, the latest findings connecting SIRT3 and oxidative stress have also provided illumination to long-standing questions regarding calorie restriction (CR) and may lead to a novel way of treating cancer.

## SIRT3: a Missing Link between Calorie Restriction and the Oxidative Stress Response

CR is one of the most robust interventions to consistently extend lifespan across species [8-10]. In mammals, this dietary regimen also results in salutary physiological changes and ameliorates a wide spectrum of diseases associated with aging. CR substantially reduces the steady state levels of oxidative stress and damage. Ostensibly, CR could slow the metabolic rate and reduce the production of ROS. However, despite the attractive simplicity of this hypothesis, it does not stand up to experimental scrutiny. Increasing evidence suggests that metabolic rate does not decrease during CR, and in some cases, even increases [11,12]. How is CR able to reduce oxidative stress without lowering metabolic rate? Does CR accomplish this feat by regulating defined molecular pathways? And, importantly, is lowered oxidative stress essential for the beneficial effects of CR? The recent work in SIRT3 has led to great strides in answering these questions.

**Figure 1.**
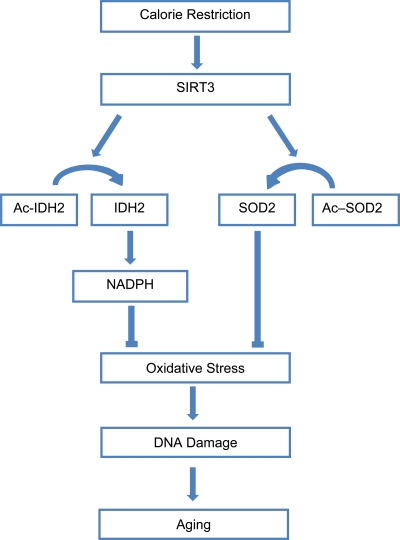
Calorie restriction activates SIRT3 to reduce oxidative stress and combat aging. Calorie restriction promotes the activity of SIRT3, which deacetylates SOD2 and IDH2, increasing the activity of these enzymes and resulting in reduced oxidative stress. Lowered oxidative stress leads to a reduced rate of aging.

Sirtuins have long been associated with CR in model organisms, so it is not surprising that this family of proteins was found to play a pivotal role in addressing the questions [13]. Previously, it had been found that the mammalian sirtuin SIRT1 is required to mediate aspects of the CR response in mice [14,15]. However, it is only with the work of Qiu *et al.* (2010) and Someya *et al.* (2010) that a solid connection between oxidative stress and sirtuins in the CR response was made [5,16]. Taking the spotlight away from SIRT1, SIRT3 was found to be essential for CR-mediated reduction in oxidative stress. Qiu *et al.* (2010) showed that the reduction in oxidative stress during CR was abolished in SIRT3-/- mice. As mentioned above, one mechanism through which SIRT3 reduces oxidative stress is by increasing the activity of SOD2 through deacetylation [5,6]. In addition to regulating SOD2, SIRT3 also reduces oxidative stress by modulating the activity of isocitrate dehydrogenase 2 (IDH2), a mitochondrial enzyme that generates the reducing agent NADPH [16]. Thus, instead of passively slowing metabolic rate, CR induces an active defense program to increase the rate at which destructive oxidizing agents are scavenged, with SIRT3 playing an essential role (Figure 1).

Does the reduction of oxidative stress have any physiological relevance with regards to the benefits of CR? Hearing loss is a hallmark of aging, and has been shown to be delayed in mice by CR. Someya *et al.* (2010) showed that the protective effect of CR on hearing loss requires reduction in oxidative stress mediated by SIRT3 [16]. When SIRT3-/- mice are placed on CR, not only is oxidative stress not reduced, but the protection against hearing loss with age is abolished as well. It was also recently shown that SIRT3 is important for protecting the heart against aging-induced hypertrophy and fibrosis, but oxidative stress has not been directly implicated [17]. It will be interesting to see if other benefits of CR can be linked to SIRT3 and oxidative stress.

## SIRT3: Balancing Cancer and Aging

**Figure 2.**
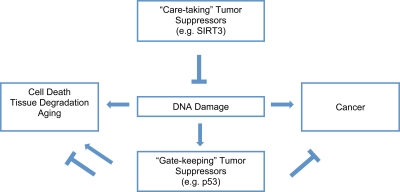
SIRT3 protects genomic stability to combat aging and cancer. DNA damage plays a causal role in aging and cancer. The damage activates “gate-keeping” tumor suppressors like p53, which protect against cancer, but can promote aging. “Care-taking” tumor suppressors like SIRT3 protect against DNA damage, which may result in protection against both aging and cancer.

Aging is a multifaceted degenerative process leading to tissue functional decline and increased incidence of cancer. One important mechanism central to aging is cumulative cellular damage, prominently DNA damage [18]. In response to DNA damage, gate-keeping tumor suppressor proteins such as p53 are activated to ensure that potentially dangerous lesions do not lead to tumorigenesis. Although the activation of gate-keeping tumor suppressor pathways has protective effects, under certain circumstances, they may contribute to the loss of tissue maintenance and drive organismal aging through the induction of apoptosis or senescence. Thus, a tenuous balance exists between aging and cancer [19-23]. Interventions that target the gate-keeping tumor suppressor pathways may slow aging at the cost of cancer or vice versa (Figure 2).

In contrast to the gate-keeping tumor suppressors, care-taking tumor suppressors maintain the stability of the genome and may simultaneously slow aging and prevent cancer. SIRT3 reduces oxidative stress, a major source of cellular and genomic damage, and may function as a gate-keeping tumor suppressor. Recently, it has been shown that deficiency of SIRT3 promotes transformation *in-vitro* in mouse embryonic fibroblasts (MEFs) [24]. Also, SIRT3-/- mice exhibit an increased risk of carcinogenesis in the mammary glands. Consistent with a causal role of oxidative stress in tumorigenesis in the absence of SIRT3, SIRT3-/- MEFs and mice have higher levels of oxidative stress and genomic instability. The level of oxidative stress could be brought down by increasing the activity of antioxidants, which suppressed transformation in SIRT3-/- MEFs. Thus, SIRT3 is emerging as a care-taking tumor suppressor that may alleviate aging and prevent cancer.

## CONCLUSION

While the role of oxidative stress in determining lifespan is still under heated debate, mounting evidence suggests that oxidative stress can affect healthspan and lead to cancer susceptibility. Combating oxidative stress can potentially have numerous therapeutic benefits. The recent findings of Qiu *et al.* (2010), Someya *et al.* (2010), Tao *et al.* (2010) and Kim *et al.* (2010) suggest that SIRT3 is involved in the cellular defense against oxidative stress [5,6,16,24]. SIRT3 could potentially be used to combat age-related decline and cancer. What remains to be seen is whether SIRT3 transgenic mice have extended lifespans and whether it is feasible to develop small molecule activators of SIRT3 to combat pathologies of aging.
